# Supplementary material for: Evidence for a Finite-Temperature Insulator
Source: Sci Rep. 2015 Aug 27;5:13503. doi: 10.1038/srep13503 (PMC4550897; doi:10.1038/srep13503)
Supplement: Supplementary Information [file srep13503-s1.pdf]

# Supplementary materials of the article - Evidence for a Finite-Temperature Insulator

M. Ovadia[1,2], D. Kalok[1], I. Tamir[1], S. Mitra[1], B. Sacepe[1,3,4] and D. Shahar[1]

[1] *Department of Condensed Matter Physics, The Weizmann Institute of Science, Rehovot 76100, Israel*

[2] *Present Address: Department of Physics, Harvard University, Cambridge, Massachusetts 02138, United States.*

[3] *Univ. Grenoble Alpes, Institut NEEL, F-38042 Grenoble, France.*

[4] *CNRS, Institut NEEL, F-38042 Grenoble, France.*

The thin film of a:InO was prepared by e-gun evaporating high purity (99.999%) $In_2O_3$ onto $SiO_2$ substrate, in an oxygen environment. The thicknesses of the film was 300 Å as measured in situ by a quartz crystal thickness monitor. The sample was lithographically defined to Hall-bar pattern.

The two probe measurements were carried out using ac lock-in techniques, by varying $T$, at the rate of 2 mK/min, while keeping $B$ constant. $V$ across the sample was kept below $10\mu$V in an effort to maintain linearity.

A more elaborate scheme was used to measure higher $R$ ($R > 10^8\ \Omega$). We started by fixing both $T$ and $B$ and, after 1 h stabilization, preformed a full $I$-$V$ scan. The $V$ source was set to

ramp smoothly between measurement points to prevent the $I$ amplifier from saturating due to capacitance induced $I$'s. A measurement was taken only after a suitable settling time (sometimes up to 10 s). Furthermore, $P = IV$ was kept low enough such that electron heating was negligible. Determining $R$ at each particular $T$ and $B$ was done by an Ohmic extrapolation of the $I$-$V$ scan to $V$=0 as demonstrated in figure 1.

To estimate leakage $I$, a null measurement (between contacts that have no continuity on the sample) was done several times. These set of measurements indicated a (resistive) isolation of at least a few $10^{12}$ $\Omega$. Pseudo-guarding (using distant wires and grounding the others) was also used to reduce mutual capacitance to a minimum and draw resistive leakages away from the input of the $I$ amplifier. The result was that the largest leakage $I$ is between the nonzero $V$ offset of the $I$ amplifier input and the "pseudo-guard", which cause a constant offset in $I$ and should not influence the $I$-$V$ slope. Drifts in that $I$ (caused by input $V$ offset drift) are probably what limit the method's accuracy because they increase with the measurement time.

In figure 2 we fit the low $T$, $B = 0.75$ T, data using an activated form, $R(T) = R_0 exp[(\frac{T_0}{T})]$. The result of our fit is plotted using the blue line in the figure, from which we obtain $T_0 = 0.918$ K and $R_0 = 0.67$ $\Omega$. The fit parameters render an activated interpretation of our data highly unlikely.

**Figure 1** **Determination of high-*R*.** $I$-$V$ characteristic curve (in log-log scale) measured at sub-threshold $V$ (in red). During the measurement $T$ and $B$ were set at 0.065 K and 1.5 T respectively. The Ohmic extrapolation of the $I$-$V$ scan is shown in dashed black, gives the $R$.

**Figure 2** **$T$ dependence of $R$.** $R$ (in log scale) vs. $T$ at $B = 0.75$ T. The solid black line represent data acquired by two-terminal measurements, while data obtained from $I$-$V$ scans are shown as triangles. The dashed lines joining the triangles are guides to the eye. The blue line is an activated-behavior fit obtained from our $T < 0.1$ K data.

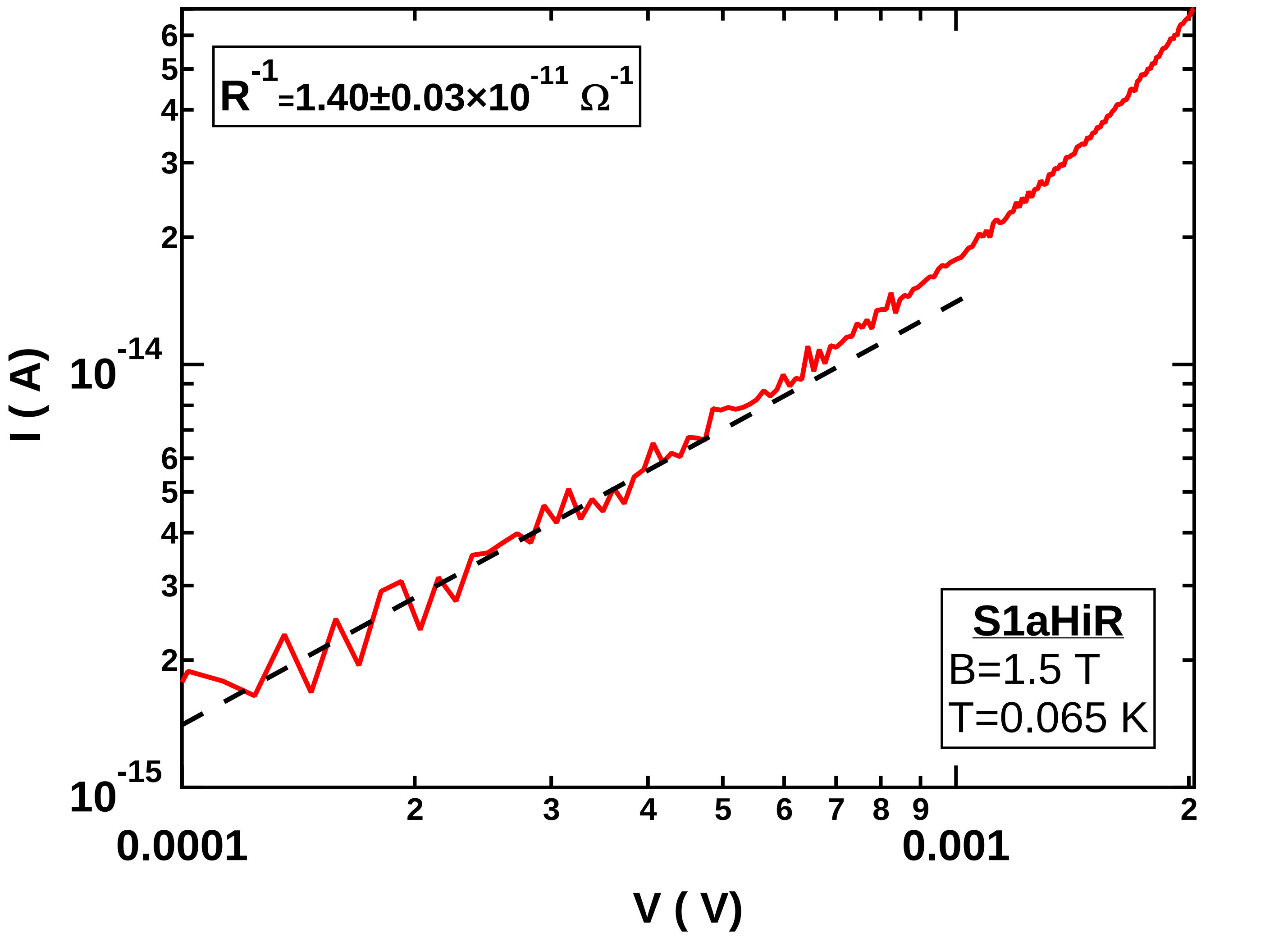

R$^{-1}$=1.40±0.03×10$^{-11}$ Ω$^{-1}$
S1aHiR
B=1.5 T
T=0.065 K
I ( A)
V ( V)
10$^{-14}$
10$^{-15}$
0.0001
0.001

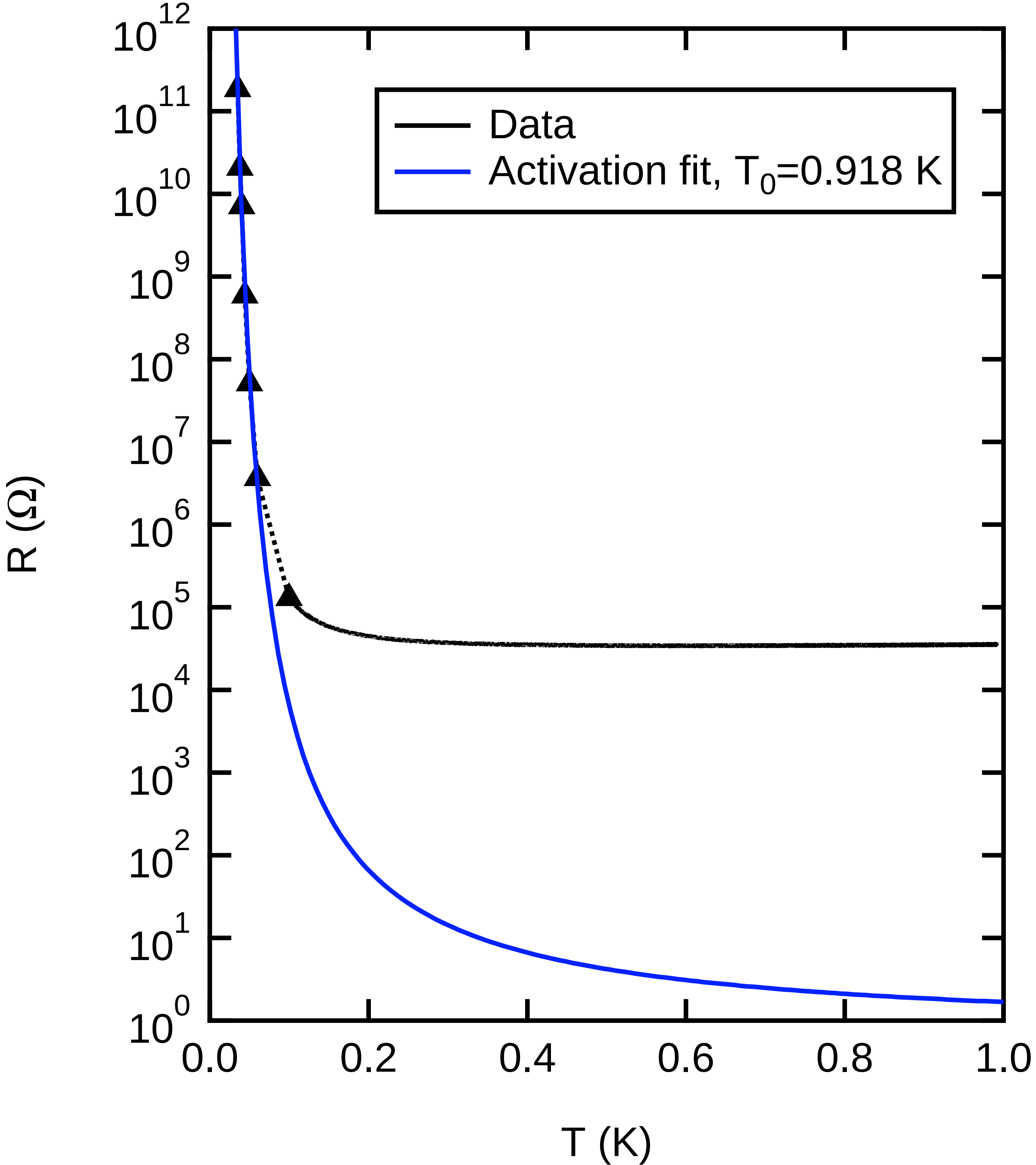

Data
Activation fit, T0=0.918 K
R (Ω)
T (K)
10^12
10^11
10^10
10^9
10^8
10^7
10^6
10^5
10^4
10^3
10^2
10^1
10^0
0.0
0.2
0.4
0.6
0.8
1.0
